# Evolutionary Analysis of Four Recombinant Viruses of the Porcine Reproductive and Respiratory Syndrome Virus From a Pig Farm in China

**DOI:** 10.3389/fvets.2022.933896

**Published:** 2022-06-24

**Authors:** Jiankui Liu, Liling Lai, Ye Xu, Yuan Yang, Jiarui Li, Chen Liu, Cuiqin Hunag, Chunhua Wei

**Affiliations:** ^1^College of Life Sciences, Longyan University, Longyan, China; ^2^Engineering Research Center for the Prevention and Control of Animal Original Zoonosis, Fujian Province University, College of Life Science, Longyan University, Longyan, China; ^3^College of Animal Science, Fujian Agriculture and Forestry University, Fuzhou, China

**Keywords:** porcine reproductive and respiratory syndrome virus (PRRSV), genome characterization, evolution, recombination, pathogenicity

## Abstract

The porcine reproductive and respiratory syndrome virus (PRRSV) is one of the most important pathogens causing substantial economic losses to the Chinese swine industry. In this study, we analyzed the complete genome sequences of four PRRSV isolates (PRRSV2/CN/SS0/2020, PRRSV2/CN/SS1/2021, PRRSV2/CN/L3/2021, and PRRSV2/CN/L4/2020) isolated from a single pig farm from 2020 to 2021. The genomes of the four isolates were 14,962–15,023 nt long, excluding the poly (A) tails. Comparative analysis of the genome sequences showed that the four isolates shared 93.2–98.1% homology and they had no close PRRSV relatives registered in the GenBank (<92%). Furthermore, PRRSV2/CN/SS0/2020 and PRRSV2/CN/SS1/2021 had characteristic 150-aa deletions (aa481+aa537-566 +aa628–747) that were identical to the live attenuated virus vaccine strain TJM-F92 (derived from the HP-PRRSV TJ). Further analysis of the full-length sequences suggests that the four isolates were natural recombinant strains between lineages 1 (NADC30-like), 3 (QYYZ-like), and 8.7 (JXA1-like). Animal experiments revealed discrepancies in virulence between PRRSV2/CN/SS0/2020 and PRRSV2/CN/L3/2021. The strain with high homology to HP-PRRSV demonstrates higher pathogenicity for pigs than the other isolate with low homology to HP-PRRSV. Taken together, our findings suggest that PRRSVs have undergone genome evolution by recombination among field strains/MLV-like strains of different lineages.

## Introduction

The porcine reproductive and respiratory syndrome (PRRS) is caused by the porcine reproductive and respiratory syndrome virus (PRRSV). It was first reported in 1987 and has spread rapidly since to become a worldwide menace (1, 2). The PRRSV genome is an ~15 kb-long positive single-stranded RNA virus that has been classified under the *Nidovirales* order and *Arteriviridae* family (https://talk.ictvonline.org/taxonomy). The viral RNA contains at least ten open overlapping reading frames (ORFs): ORF1a, ORF1b, ORF2a, ORF2b, ORF3, ORF4, ORF5a, and ORF5–ORF7 (3–6). Among these ORFs, ORF1a and ORF1b comprise almost three-quarters of the viral genome and encode at least 16 nonstructural proteins (such as nsp1α, nsp1β, nsp2-6, nsp2TF, nsp2N, nsp7α, nsp7β, and nsp8–12), whereas other ORFs located at the 3' terminus code for eight structural proteins: GP2, E, GP3, GP4, GP5a, GP5, M, and N, respectively (3, 5, 7, 8).

All PRRSV strains isolated to date have been classified into two major genotypes, namely, the European (type 1) and North American (type 2) genotypes, with type 2 PRRSV being predominant in China since its initial report there in 1996 (9, 10). PRRS is considered one of the most important infectious diseases in Chinese swineherds as it causes severe economic losses in the swine industry every year. Currently, the Chinese type 2 PRRSV strains can be classified into different lineages, including lineage 1 (NADC30-like), 3 (QYYZ-like), 5 (VR2332-like), and 8 (JXA1-like and CH-1a-like) based on phylogenetic analysis of ORF5 sequences (11, 12). The diversity of the Chinese type 2 PRRSV has been increasing due to recombination events among the different PRRSV lineages since the emergence of NADC30-like PRRSVs in China in 2012 (12–23). In the present study, we identify four recombinant PRRSV strains circulating in a swine farm in Fujian province, China. Phylogenetic and molecular evolutionary analyses indicated that these strains evolved from natural recombination among the NADC30-, QYYZ-, and JXA1-like variants. To further understand the four strains, we genetically characterized the complete genomes of the viruses.

## Materials and Methods

### Sample Collection and Viral Isolation

From 2020 to 2021, a severe reproductive and respiratory disease was observed in pigs from four independent pens in a swine farm in Fujian province, China. This farm created a PRRS-positive stable herd by inoculating 0.05 dose/pig of MLV TJM-F92 vaccine (derived from the HP-PRRSV TJ) from 2018. The affected pigs from the two pens exhibited high fever (40.3–41.8°C), severe respiratory syndrome, and high morbidity (30%) and mortality (20%), whereas those from the other two pens exhibited fever, severe respiratory syndrome and reproductive problems, and especially high abortion rate (10%) was observed in the sows. PRRSV was detected using an RT-PCR kit (Beijing Anheal Laboratories Co., Ltd., Beijing, China) according to the manufacturer's instructions. PRRSV was positive when the CT value was ≤30. MARC-145^CD163^ cell lines (a stable cell line highly expressing porcine CD163) were used for PRRSV isolation from the positive samples (12). The tissues (lung, lymph nodes, and serum samples) were homogenized with DMEM containing antibiotics and antimycotics to obtain a 50% (w/v) suspension. After freeze-thawing thrice, the samples were centrifuged at 10,000 × g for 10 min and the supernatants were sieved through a 0.22-μm filter. MARC-145^CD163^ cells were mixed with 100 μL of supernatant and incubated at 37 °C with 5% CO_2_ for 3–5 days. PRRSVs were passaged three times in MARC-145^CD163^ cells for subsequent analysis after being plaque-purified three times.

### RNA Isolation and RT-PCR

RNA was extracted from the positive tissues (lungs, serum, and lymph nodes) and the virus using a Viral RNA Mini Kit (TIANGEN, Beijing, China) according to the manufacturer's instructions. cDNA was generated using the HiScript^®^ III 1st Strand cDNA Synthesis Kit (Vazyme Biotech Co., Ltd, Nanjing, China) following the supplier's instructions. The full-length viral genomes were amplified using six viral-specific primers (Supplementary Table 1). Subsequently, we confirmed the complete genome using other PCR primers as described previously (24, 25). The purified PCR products were cloned into the pEASY^®^-Blunt Simple Cloning Vector (TransGen Biotech, Beijing, China). To determine a consensus sequence of each fragment, at least three recombinant clones were sent to the Ruibo Life Technologies Corporation (Beijing, China) for sequencing by using the Sanger approach in both directions for each fragment.

### Complete Genomic Sequence and Recombination Analysis

Forty-four representative PRRSV strains available in GenBank, including the type 1 and type 2 strains, were used for the comparative sequence analysis in this study (Table 1). Multiple sequence alignments and genome analyses were performed using the MEGA 7.0 software and the DNAStar 7.0 package. Phylogenetic trees were constructed using the neighbor-joining method in MEGA 7.0 and bootstrap confidence values from 1,000 replicates.

**Table 1:** Representative PRRSV strains used in this study.

**No**.	**Name**	**GenBank accession no**.	**Origin**	**No**.	**Name**	**GenBank accession no**.	**Origin**
1	JXwn06	EF641008	China	23	FJZ03	KP860909	China
2	TJ	EU860248	China	24	MN184A	DQ176019	U.S.A
3	JXA1	EF112445	China	25	MN184B	DQ176020	U.S.A
4	HUN4	EF635006	China	26	HENNAN-XINX	KF611905	China
5	JXA1 P80	FJ548855		27	MN184C	EF488739	U.S.A
6	MLV-like TJbd14-1	KP742986	China	28	FJY04	KP860910	China
7	CH-1a	AY032626	China	29	FJYR	KT804696	China
8	CH-1R	EU807840	China	30	FJM4	KY412888	China
9	HB-1(sh)/2002	AY150312	China	31	JX143	EU708726	China
10	HB-2(sh)/2002	AY262352	China	32	HLJA1	KT351739	China
11	VR-2332	U87392	China	33	NT1	KP179402	China
12	NT1	KP179402	China	34	FJZH	KP998478	China
13	PA8	AF176348	Canada	35	FJSD	KP998474	China
14	BJ-4	AF331831	China	36	1105-GD-GL	KR612137	China
15	RespPRRS MLV	AF066183	U.S.A	37	110102-GD-ST	KR018789	China
16	GD-KP	KU978619	China	38	YJ1-10	KC282627	China
17	GM2	JN662424	China	39	140116-GD-YWC	KR018799	China
18	FJFS	KP998476	China	40	140520-GD-SXB	KR018784	China
19	QYYZ	JQ308798	China	41	GDsg	KX621003	China
20	NADC30	JN654459	U.S.A	42	GXBS06-2012	KC617956	China
21	FJY04	KP860910	China	43	LV	M96262	Netherlands
22	CHsx1401	KP861625	China	44	MLV TJM F-92		China

Potential recombination within the whole genome sequences was determined using seven methods (RDP, BootScan, GENECONV, Chimera, Maxchi, SiScan, and 3Seq) *via* the recombination detection program 4.10 (RDP 4.10) (26). A recombination event was identified when at least five of the seven methods reported recombination signals in RDP 4.10 with the highest acceptable *p* of 0.05 (27). The possible recombination event was further confirmed using the SIMPLOT software (version 3.5.1) with a 200-bp window width and a 20-bp step size (28).

### Challenge Experiment

To evaluate the pathogenicity of the recombinant strains, two strains PRRSV2/CN/SS0/2020 (98.3% homology with JXA1) and PRRSV2/CN/L3/2021 (86.3% homology with JXA1) were selected for animal experiments. Fifteen 4-week-old pigs confirmed to be free of PRRSV and PCV2 were randomly divided into 3 groups (5 pigs/group). The pigs in group 1 were intranasally administered with 2 mL of PRRSV2/CN/SS0/2020 containing 2 × 10^5^ TCID50 and those in group 2 were intranasally administered with 2 mL of PRRSV2/CN/SS0/2020 containing 2 × 10^5^ TCID50. The pigs in the negative control group were inoculated with 2 mL of Dulbecco's Modified Eagle Medium (DMEM). Rectal temperature was recorded daily from 0 to 14 days post-challenge (dpc). Serum was collected at 0, 4, 7, 11, and 14 dpc to detect PRRSV-specific antibodies using PRRSV using ELISA kit (IDEXX Laboratories Inc., Westbrook, ME, USA). Viral load in the sera of each group was detected by an IFA-microtitration infectivity assay as previously described (29). All animals were euthanized at 14 dpc for necropsy. In addition, lung tissues of pigs were collected at necropsy and fixed in 10% neutral-buffered formalin and routinely processed for histological examination. The procedures of animal handling and experimentation performed in this study were approved by the Longyan University Animal Ethics Committee (Permit number: permit no.LY20210010X).

### Data Analysis

Statistical analysis in this study was performed using a one- or two-way ANOVA analysis of variance in GraphPad Prism software (version 6.0), and the results were considered statistically significant when *p* < 0.05.

## Results

### Complete Genome Sequence Analysis of PRRSV

Four strains of PRRSV were isolated using MARC-145^CD163^ cells and designated as PRRSV2/CN/SS0/2020 (Accession number: ON365556), PRRSV2/CN/SS1/2021 (Accession number: ON093974), PRRSV2/CN/L3/2021 (Accession number: OL416130), and PRRSV2/CN/L4/2020 (Accession number: OL422822). Typical PRRSV CPE characterized by cell fusion and shedding was observed in MARC-145CD^163^ cells (Supplementary Figure 1). The genomes of the four isolates were 14,962–15,023 nt in length, excluding the poly (A) tails at the 3' end. Comparative analyses of genome sequences showed that the four isolates shared 93.2–98.1% homology and had a 83.7–86.8% identity with VR2332-like PRRSVs (VR-2332, RespPRRSV MLV, and BJ-4), 86.1–91.5% with JXA1-like PRRSVs (JXA1, HuN4, and TJd14-1), 81.9–86.6% with NADC30-like PRRSVs (NADC30, FJZ03, and Chsx1401), 82.9–86.5% with QYYZ-like PRRSVs (QYYZ, GM2, and FJFS), and only 59.2–59.8% with LV (Supplementary Table 2).

To evaluate the genomic characteristics of the four PRRSV isolates, each region of the genomes of the four strains was further compared with four viruses from different lineages, including NADC30-like strains, QYYZ-like strains, VR2332-like strains (VR-2332 and BJ-4), JXA1-like strains, and the LV (the prototype of type 1 PRRSV) strain (Table 2). The 5'-UTR of the four PRRSV isolates was 188–191 nt long and they had an identity of 87.3–91.5% with NADC30-like strains, 93.7–95.7% with QYYZ-like strains, 89.4–91.0% with VR2332-like strains, 97.4–98.4% with JXA1-like strains, and 61.5–62.2% with LV. The 3'-UTR of the four PRRSVs was found to be ~145–154 nt long, excluding the poly (A) tail, and their sequence was 86.1–89.9% homologous with that of the NADC30-like strains, 89.4–92.1% with that of the QYYZ-like strains, 90.7–91.4% with that of the VR2332-like strains, 85.3–88.7% with that of the JXA1-like strains, and 74.3–75.2% with that of LV (Table 2).

**Table 2:** Sequence distance of four isolates in this study with reference strains.

	**VR2332**	**BJ-4**	**JXA1**	**HuN4**	**TJbd14-1**	**NADC30**	**CHsx1401**	**FJZ03**	**QYYZ**	**FJFS**	**GM2**	**LV**
	% identity toPRRSV2/CN/SS1/2021 /% identity toPRRSV2/CN/L3/2021/% identity to PRRSV2/CN/L4/2020/ PRRSV2/CN/SS0/2020											
**Nucleotides**												
5' UTR	89.4/91.0/ 90.4/90.5	89.4/91.0/ 90.4/90.5	97.4/98.4/ 97.9/98.4	97.4/98.4/ 97.9/98.4	97.4/97.9/ 97.9/98.4	89.9/91.5/ 91.0/91.0	87.3/ 88.8/88.3/88.4	88.9/90.4/ 89.9/89.9	93.7/94.1/ 94.7/94.7	95.2/94.1/ 95.7/95.2	93.7/94.1/ 94.7/94.7	62.0/62.0/ 61.5/61.7
ORF1a	53.2/82.7/ 82.5/84.7	83.1/82.4/ 82.3/84.5	89.6/84.7/ 84.6/92.2	89.6/84.7/ 84.6/92.3	89.6/84.9/ 84.9/92.4	83.2/ 86.6/86.7/82.5	82.1/85.1/ 85.2/81.5	82.1/85.9/ 86.0/81.8	81.2/80.0/ 79.9/82.4	81.9/80.8/ 80.8/83.3	80.9/79.8/ 79.7/82.0	55.1/55.1/ 55.1/55.1
ORF1b	88.4/88.4/ 88.5/88.5	88.5/88.5/ 88.5/88.6	92.9/93.0/ 93.2/93.1	92.8/92.9/ 93.1/93.1	92.7/92.7/ 93.0/93.0	86.3/86.3/ 86.4/86.4	85.7/85.7/ 85.8/85.8	85.9/85.9/ 86.0/86.0	88.1/88.2/ 88.4/88.2	87.6/87.7/ 87.7/87.8	83.3/83.3/ 83.4/88.3	63.2/63.2/ 63.2/63.1
ORF2-7	87.8/88.0/ 88.8/88.3	87.6/87.8/ 88.6/88.1	86.6/86.6/ 87.3/87.1	86.9/86.9/ 87.5/87.4	86.7/86.7/ 87.4/87.2	85.7/85.4/ 86.4/85.6	85.4/85.1/ 86.1/85.3	85.2/84.9/ 85.6/85.1	89.6/89.5/ 90.1/90.0	90.0/89.9/ 90.7/90.3	89.2/89.1/ 89.7/89.6	65.2/65.2/ 65.3/65.2
3' UTR	90.7/90.7/ 91.4/92.7	90.7/90.7/ 91.4/92.7	87.3/85.3/ 86.0/88.7	87.3/85.3/ 86.0/88.7	87.3/85.3/ 86.0/88.7	88.7/87.4/ 89.4/89.4	87.4/86.1/ 88.1/88.1	89.2/86.5/ 86.5/89.9	90.7/92.1/ 90.7/92.1	89.4/90.7/ 89.4/90.7	89.4/90.7/ 89.4/90.7	75.2/75.2/ 74.3/74.3
**aa**												
NSP1α	95.2/96.4/ 95.8/97.0	95.2/96.4/ 95.8/97.0	94.6/95.8/ 95.2/97.6	94.6/95.8/ 95.2/97.6	94.6/95.8/ 95.2/97.6	95.2/96.4/ 95.8/95.8	92.8/94.8/ 93.4/95.8	92.8/93.4/ 92.8/94.0	96.4/97.1/ 96.4/97.6	92.8/93.4/ 92.8/95.2	96.4/97.0/ 96.4/83.2	65.1/65.1/ 64.5/65.7
NSP1β	81.6/82.5/ 82.5/82.5	81.1/82.0/ 82.0/82.0	93.5/94.0/ 94.0/94.5	94.0/94.5/ 94.5/94.9	93.1/93.5/ 93.5/94.0	74.2/74.2/ 75.6/74.7	72.4/72.4/ 73.7/72.8	74.2/73.7/ 75.1/74.2	82.5/83.4/ 83.4/83.4	88.9/89.4/ 89.4/89.9	82.9/83.4/ 83.4/83.9	41.3/41.8/ 41.8/41.8
NSP2	71.0/69.4/ 69.2/74.1	70.4/68.9/ 68.7/73.5	85.3/68.2/ 67.8/91.6	85.2/67.6/ 67.4/91.6	86.9/65.6/ 65.5/93.8	70.6/82.9/ 83.6/68.7	68.7/80.1/ 80.4/67.3	70.1/82.1/ 82.9/68.0	69.0/63.8/ 63.4/72.0	70.6/66.5/ 66.7/74.2	68.4/63.8/ 63.4/71.4	28.9/32.0/ 32.2/27.9
NSP3	91.5/91.7/ 91.5/92.8	91.5/91.7/ 91.5/92.8	92.4/91.3/ 91.5/94.4	92.6/91.5/ 91.7/94.4	92.6/91.7/ 91.9/95.1	90.1/90.6/ 90.4/89.7	93.9/96.0/ 95.3/92.4	94.4/95.1/ 94.4/93.3	82.2/82.7/ 82.4/88.1	82.2/82.7/ 82.4/88.1	82.2/82.7/ 82.4/88.1	58.2/58.2/ 58.0/58.7
NSP4	94.6/94.6/ 94.6/94.6	94.6/94.6/ 94.6/94.6	96.6/96.6/ 96.6/96.6	96.6/96.6/ 96.6/96.6	96.1/96.1/ 96.1/96.1	96.1/96.1/ 96.1/96.1	93.1/93.1/ 93.1/93.1	92.6/92.6/ 92.6/92.6	93.1/93.1/ 93.1/93.1	92.6/92.6/ 92.6/92.6	93.6/93.6/ 93.6/93.6	62.1/62.1/ 62.1/62.1
NSP5	88.8/88.2/ 88.8/88.8	89.4/88.8/ 89.4/89.4	91.2/91.8/ 91.8/91.2	91.2/91.8/ 91.8/91.2	91.2/91.8/ 91.8/91.2	91.2/91.8/ 91.8/91.2	88.8/89.4/ 90.0/88.8	90.6/90.6/ 91.2/90.6	88.2/88.2/ 88.8/88.2	87.6/87.6/ 88.2/87.6	87.6/87.6/ 88.2/87.6	71.2/71.8/ 72.4/71.2
NSP6	87.5/87.5/ 87.5/87.5	87.5/87.5/ 87.5/87.5	93.8/93.8/ 93.8/93.8	93.8/93.8/ 93.8/93.8	93.8/93.8/ 93.8/93.8	87.5/87.5/ 87.5/87.5	81.2/81.2 /81.2/81.2	87.5/87.5/ 87.5/87.5	93.8/93.8/ 93.8/93.8	93.8/93.8/ 93.8/93.8	93.8/93.8/ 93.8/93.8	75.0/75.0/ 75.0/75.0
NSP7	88.0/87.3/ 87.3/88.4	86.9/86.9/ 86.1/87.3	92.3/93.1/ 92.3/92.7	92.3/93.1/ 92.3/92.7	92.3/93.1/ 92.3/92.7	84.2/84.6/ 83.8/84.6	83.0/83.4/ 82.6/83.4	83.4/83.8/ 83.0/83.8	88.8/89.6/ 88.8/89.2	89.2/90.0/ 89.2/89.6	88.0/88.0/ 87.3/88.4	47.8/48.2/ 47.8/48.2
NSP8	95.6/97.8/ 97.8/95.6	95.6/97.8/ 97.8/95.6	95.6/97.8/ 97.8/95.6	95.6/97.8/ 97.8/95.6	95.6/97.8/ 97.8/95.6	91.1/93.3/ 93.3/91.1	88.9/91.1/ 91.1/88.9	91.1/93.3/ 93.3/91.1	95.6/97.8/ 97.8/95.6	91.1/93.3/ 93.3/91.1	95.6/97.8/ 97.8/95.6	65.9/68.2/ 68.2/65.9
NSP9	96.9/97.2/ 97.2/97.0	96.6/96.9/ 96.9/96.7	98.1/98.4/ 98.4/98.3	98.0/98.3/ 98.3/98.1	98.1/98.4/ 98.4/98.3	96.6/96.9/ 96.9/96.7	95.6/95.9/ 95.9/95.8	96.0/96.3/ 96.3/96.1	96.4/96.7/ 96.7/96.6	95.2/95.2/ 95.3	96.4/96.7/ 96.6	74.8/74.6/ 74.6/74.6
NSP10	95.5/95.5/ 95.5/95.5	95.5/95.5/ 95.5/95.5	98.2/98.2/ 98.2/98.2	98.9/98.9/ 98.9/98.9	98.0/98.0/ 98.0/98.0	95.0/95.0/ 95.0/95.0	94.8/94.8/ 94.8/94.8	94.6/94.6/ 94.6/94.6	97.3/97.3/ 97.3/97.3	93.7/93.7/ 93.7/93.7	95.7/95.7/ 95.7/95.7	64.9/64.9/ 64.9/64.9
NSP11	92.8/92.8/ 92.8/92.8	93.7/93.7/ 93.7/93.7	96.9/96.9/ 96.9/96.9	96.9/96.9/ 96.9/96.9	96.9/96.9/ 96.9/96.9	95.5/95.5/ 95.5/95.5	93.3/93.3/ 93.3/93.3	94.2/94.2/ 94.2/94.2	93.3/93.3/ 93.3/93.3	92.8/92.8/ 92.8/92.8	93.7/93.7/ 93.7/93.7	76.6/76.6/ 76.6/76.6
NSP12	92.2/91.5/ 92.2/92.2	92.2/91.5/ 92.2/92.2	95.4/94.8/ 95.4/95.4	95.4/94.8/ 95.4/95.4	95.4/94.8/ 95.4/95.4	92.2/91.5/ 92.2/92.2	91.5/90.8/ 91.5/91.5	93.5/92.8/ 93.5/93.5	92.8/92.2/ 92.8/92.8	93.5/94.1/ 93.5/93.5	93.5/92.8/ 93.5/93.5	46.3/46.3/ 46.3/46.3
ORF2a/GP2	91.0/90.2/ 91.0/91.4	90.2/89.5/ 90.2/90.6	87.9/87.9/ 88.7/88.3	87.5/87.5/ 88.3/87.9	88.3/88.3/ 89.1/88.7	89.5/87.5/ 88.3/88.7	91.8/89.8/ 90.6/91.0	88.7/87.5/ 87.5/87.9	93.0/93.0/ 93.8/93.4	89.5/89.5/ 90.2/89.8	92.6/92.6/ 93.4/93.0	61.4/61.8/ 62.2/61.8
ORF2b/E	91.8/93.2/ 93.2/91.8	90.4/91.8/ 91.8/90.4	91.8/90.4/ 90.4/91.8	91.9/90.4/ 90.4/91.9	91.8/90.4/ 90.4/91.8	91.8/90.4/ 90.4/91.8	89.0/87.7/ 87.7/89.0	91.8/90.4/ 90.4/91.8	94.5/93.2/ 93.2/94.5	82.2/80.8/ 80.8/82.2	94.5/93.2/ 93.2/94.5	71.8/71.4/ 71.4/71.8
ORF3/GP3	86.6/87.4/ 86.6/87.0	87.0/87.8/ 87.0/87.4	84.6/85.0/ 84.3/85.0	85.0/85.4/ 84.6/85.4	84.6/85.0/ 84.3/85.0	84.3/84.3/ 83.5/83.5	84.6/84.6/ 83.9/83.9	81.9/82.3/ 81.1/81.5	88.2/88.2/ 87.4/87.8	90.2/90.2/ 89.4/89.8	87.4/87.0/ 86.6/87.4	56.5/57.3/ 56.1/56.5
ORF4/GP4	89.3/89.9/ 90.4/89.3	89.3/89.9/ 90.4/89.3	87.1/87.6/ 88.2/87.1	89.3/89.9/ 90.4/89.3	89.3/89.9/ 90.4/89.3	87.1/87.6/ 88.2/87.1	86.0/86.5/ 87.1/86.0	86.0/86.5/ 87.1/86.0	87.6/88.2/ 88.8/87.6	90.4/90.4/ 91.0/90.4	87.1/87.6/ 88.2/87.1	69.7/70.2/ 69.7/69.7
ORF5/GP5	81.0/81.5/ 81.0/82.5	80.5/81.0/ 80.5/82.0	82.5/83.1/ 81.5/83.5	82.5/83.0/ 81.5/83.5	82.5/83.0/ 81.5/83.5	84.0/84.5/ 83.0/85.0	83.5/83.5/ 84.0/84.5	82.5/82.5/ 82.0/83.5	91.5/92.0/ 92.0/93.0	91.0/91.5/ 91.5/92.5	90.0/90.5/ 90.5/91.5	58.2/57.1/ 57.1/58.7
ORF5a	78.3/78.3/ 78.3/78.3	78.3/78.3/ 78.3/78.3	71.7/71.7/ 71.7/71.7	71.7/71.7/ 71.7/71.7	71.7/71.7/ 71.7/71.7	76.1/76.1/ 76.1/76.1	76.1/76.1/ 76.1/76.1	78.3/78.3/ 78.3/78.3	89.1/89.1/ 89.1/89.1	89.1/89.1/ 89.1/89.1	91.3/91.3/ 91.3/91.3	46.5/46.5/ 46.5/46.5
ORF6/M	94.3/94.3/ 94.8/94.3	94.3/94.3/ 94.8/94.3	93.1/93.1/ 94.3/93.1	93.1/93.1/ 94.3/93.1	93.1/93.1/ 94.3/93.1	93.1/93.1/ 93.7/93.1	93.1/93.1/ 94.8/93.1	93.7/93.7/ 94.3/93.7	95.4/95.4/ 97.1/95.4	96.0/96.0/ 97.1/96.0	95.4/95.4/ 96.6/95.4	78.0/78.0/ 78.0/78.0
ORF7/N	92.7/91.1/ 91.1/91.9	92.7/91.1/ 91.1/91.9	92.7/91.1/ 91.1/91.9	92.7/91.1/ 91.1/91.9	91.9/90.2/ 90.2/91.1	89.4/88.6/ 92.7/90.2	84.6/83.7/ 87.8/85.4	88.6/87.8/ 91.9/89.4	90.2/88.6/ 91.7/92.7	90.3/88.6/ 93.5/90.2	86.2/84.6/ 87.8/88.6	62.0/60.8/ 63.3/61.7

*aa, Amino acids*.

ORF1a and ORF1b encode the non-structural proteins (Nsp) of PRRSV. A nucleotide sequence comparison of ORF1 showed that in the four strains, ORF1b was relatively conserved as compared to ORF1a. Of all the Nsp sequences, the most variable ones were from Nsp1β and Nsp2 within ORF1a, which had 72.4–94.9% and 63.4–86.9% amino acid identity with the reference strains, respectively (Table 2).

Open reading frames 2a to 7 encode the PRRSV structural proteins. Nucleotide sequence comparison of this region showed that the four strains shared 95.2–99.1% nucleotide identity, 84.9–86.4% identity with NADC30-like strains, 89.1–90.7% identity with QYYZ-like strains, 87.6–88.8% identity with VR2332-like strains, and 86.6–87.5% identity with JXA1-like strains. In contrast, these ORF regions only shared 65.2–65.3% identity with LV.

### Phylogenetic Analysis of PRRSV

Four respective phylogenetic trees were constructed based on the full-length genome sequences, Nsp2, ORF2-7, and ORF5 nucleotide sequences of the four PRRSV isolates and 27 representative strains of type 2 PRRSV.

**Figure 1:**
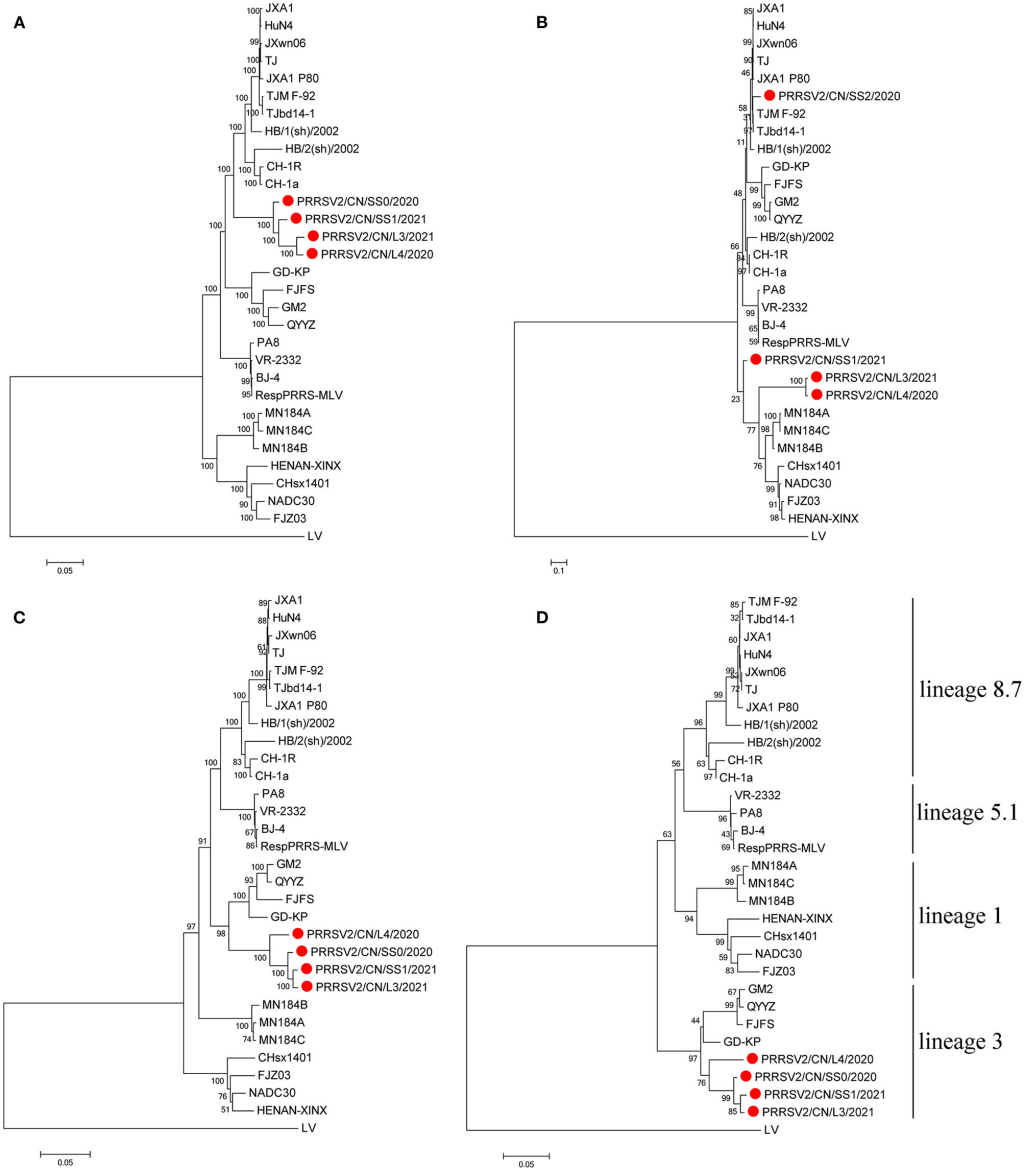
Phylogenetic tree based on the full length **(A)**, Nsp2 **(B)**, ORF2-7 **(C)**, and ORF5 **(D)** of the four PRRSV strains from this study and reference strains. The four PRRSV strains isolated in this study are labeled with a red circle. The reliability of the tree was evaluated by bootstrapping using Mega 7.0 with 1000 replicates.

Interestingly, the tree based on the full-length genome sequences showed that the four PRRSVs formed a separate branch that was in the middle of lineages 3 and 8.7, which are represented by QYYZ and JXA1, respectively (Figure 1A). The tree based on the ORF2-7 or ORF5 sequences indicates that the four strains were grouped in lineage 3, together with the QYYZ-like strains (Figures 1C,D). The tree constructed based on Nsp2 indicates that PRRSV2/CN/L3/2021 and PRRSV2/CN/L4/2020 were clustered into lineage 1, together with the NADC30-like strains, and PRRSV2/CN/SS0/2020 was clustered into lineage 8.7, together with the JXA1-like strains; however, PRRSV2/CN/SS1/2021 was in a separate branch (Figure 1B).

### Amino Acid Analysis of NSP2 and ORF5

**Figure 2:**
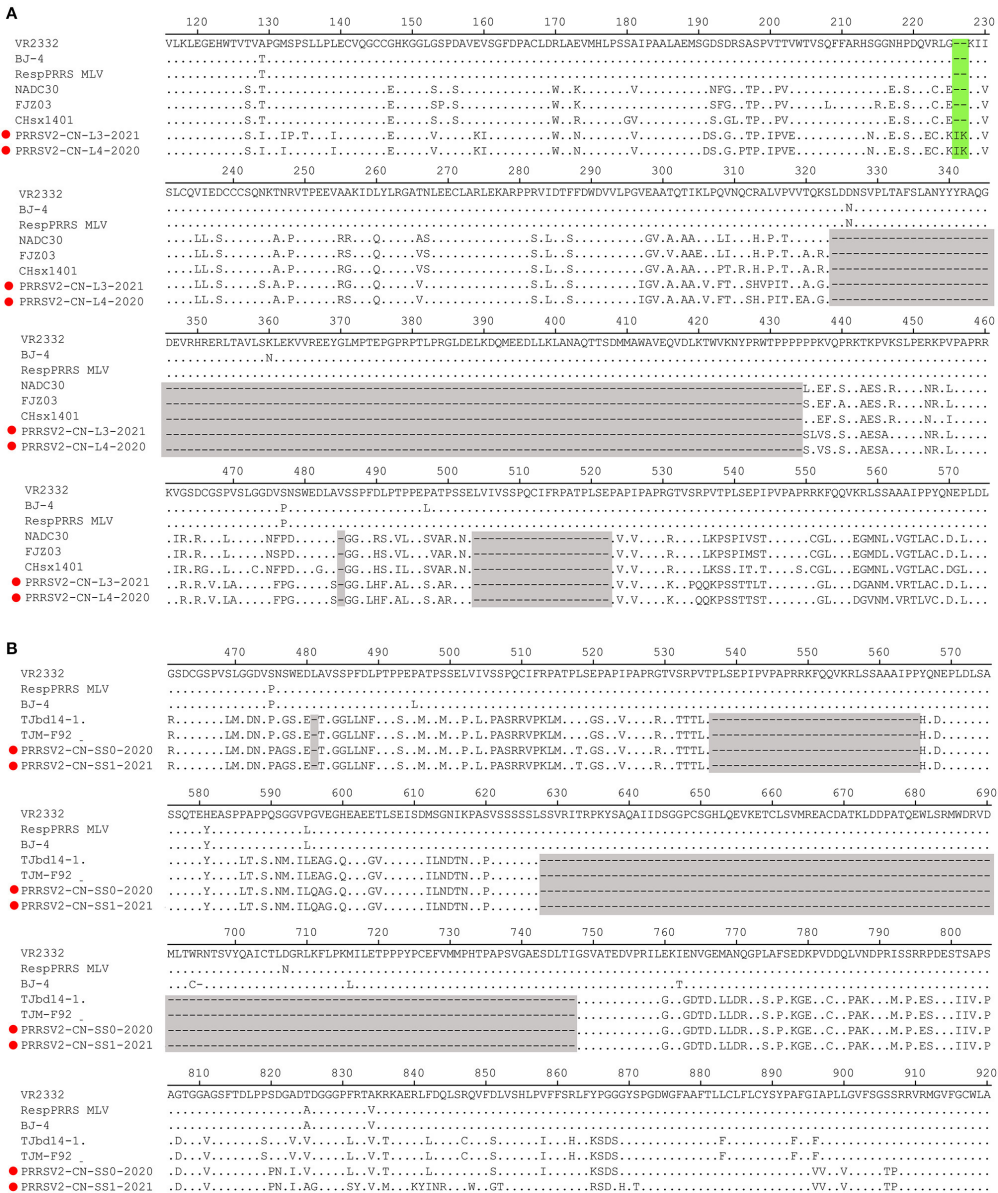
Amino acids alignment for Nsp2 of the four PRRSV isolates with representative strains. **(A)** The 131-aa discontinuous deletions (aa324–434, aa485, and aa504–522) highlighted in gray boxes show the deletion signature of PRRSV2/CN/L3/2021, PRRSV2/CN/L4/2021, and NADC30-like strains. Additional 2-aa insertion in PRRSV2/CN/L3/2021 and PRRSV2/CN/L4/2021 is marked in the green box. **(B)** The 150-aa discontinuous deletions (aa481+aa537-566+aa628–747) highlighted in gray boxes show the deletion signature of PRRSV2/CN/SS0/2020, PRRSV2/CN/SS1/2021, and MLV TJM-F92 strain.

The Nsp2-coding region is recognized as one of the most variable proteins with different deletions and insertions. Therefore, Nsp2 is often used to analyze the genetic variations and molecular epidemiology of PRRSV. As shown in Figure 2, two isolates, namely PRRSV2/CN/L3/2021 and PRRSV2/CN/L4/2020, had a unique discontinuous deletion of 131-aa in the Nsp2-coding region, which was identical to that of the NADC30 and NADC30-like strains. Meanwhile, PRRSV2/CN/L4/2020 had an additional 1-aa deletion at the 15th aa. Interestingly, both PRRSV2/CN/L3/2021 and PRRSV2/CN/L4/2020 had two additional 2-aa insertions at amino acid positions 224–225 (Figure 2A). Notably, PRRSV2/CN/SS0/2020 and PRRSV2/CN/SS1/2021 had characteristic 150-aa deletions (aa481+aa537-566+aa628–747) that were identical to those of the live attenuated virus vaccine strain TJM-F92 (derived from the HP-PRRSV TJ) (30) and TJbd14–1 (13), which is an MLV-like strain that evolved from the HP-PRRSV vaccine virus TJM-F92 (Figure 2B). Notably, comparative sequence analysis showed that PRRSV2/CN/SS0/2020 and PRRSV2/CN/SS1/2021 exhibited the highest nucleotide similarity (89.5–95.3%) and amino acid similarity (87.0–94.0%) with TJbd14-1 and TJM-F92.

**Figure 3:**
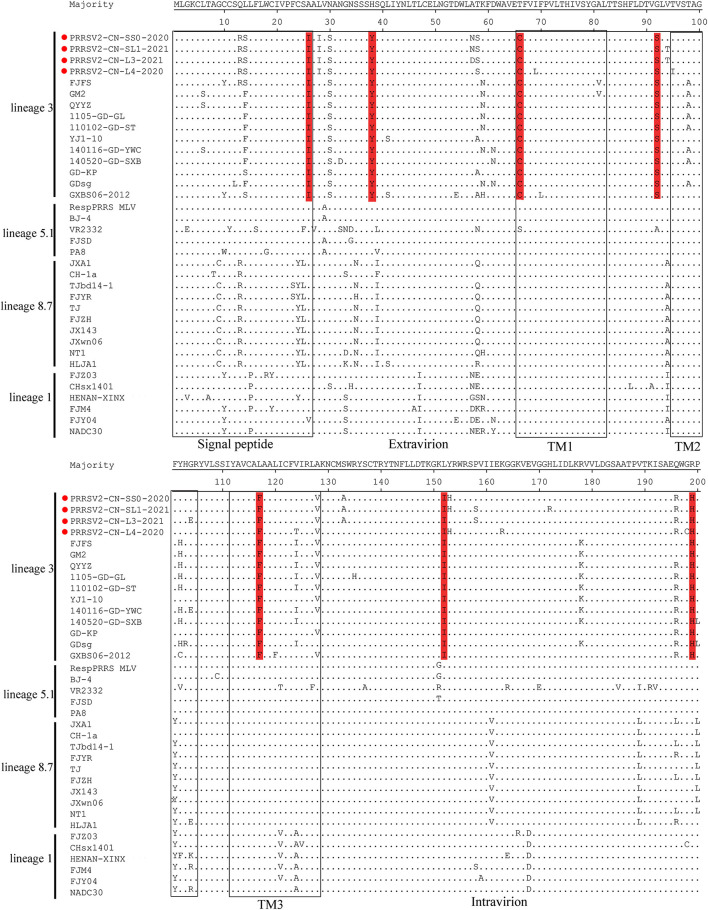
Analysis and comparison of amino acid mutations in GP5 compared to reference viruses. The signal peptide and transmembrane (TM) domains in GP5 are highlighted by rectangles. Important amino acid changes between four strains (red cicrlce) and QYYZ-like PRRSVs in GP5 are indicated in red.

The GP5 protein encoded by ORF5 is the most variable structural protein of the virus. At least six antigenic regions (ARs) were reported within GP5 (AR1-15, AR27-35, AR37-51, AR149-156, AR166-181, and AR192-200) (31, 32). Three ARs (AR27-35, AR37-51, and AR192-200) at the N-terminus of the four strains were the most similar to the QYYZ-like strains but differed from the JXA1-like, VR-2332-like, and NADC30-like strains. Additionally, seven unique amino acids distributed in GP5 were only identified in QYYZ-like strains and the four strains (I^26^, Y^38^, C^66^, S^92^, F^117^, I^152^, and H^199^) (Figure 3). Studies have shown that the 13th and 151st amino acids of GP5 are related to the virulence of the virus (33). In the present study, four strains had both R^13^Q and R^151^G mutation as compared with the VR2332 strain.

### Recombination Analysis

**Figure 4:**
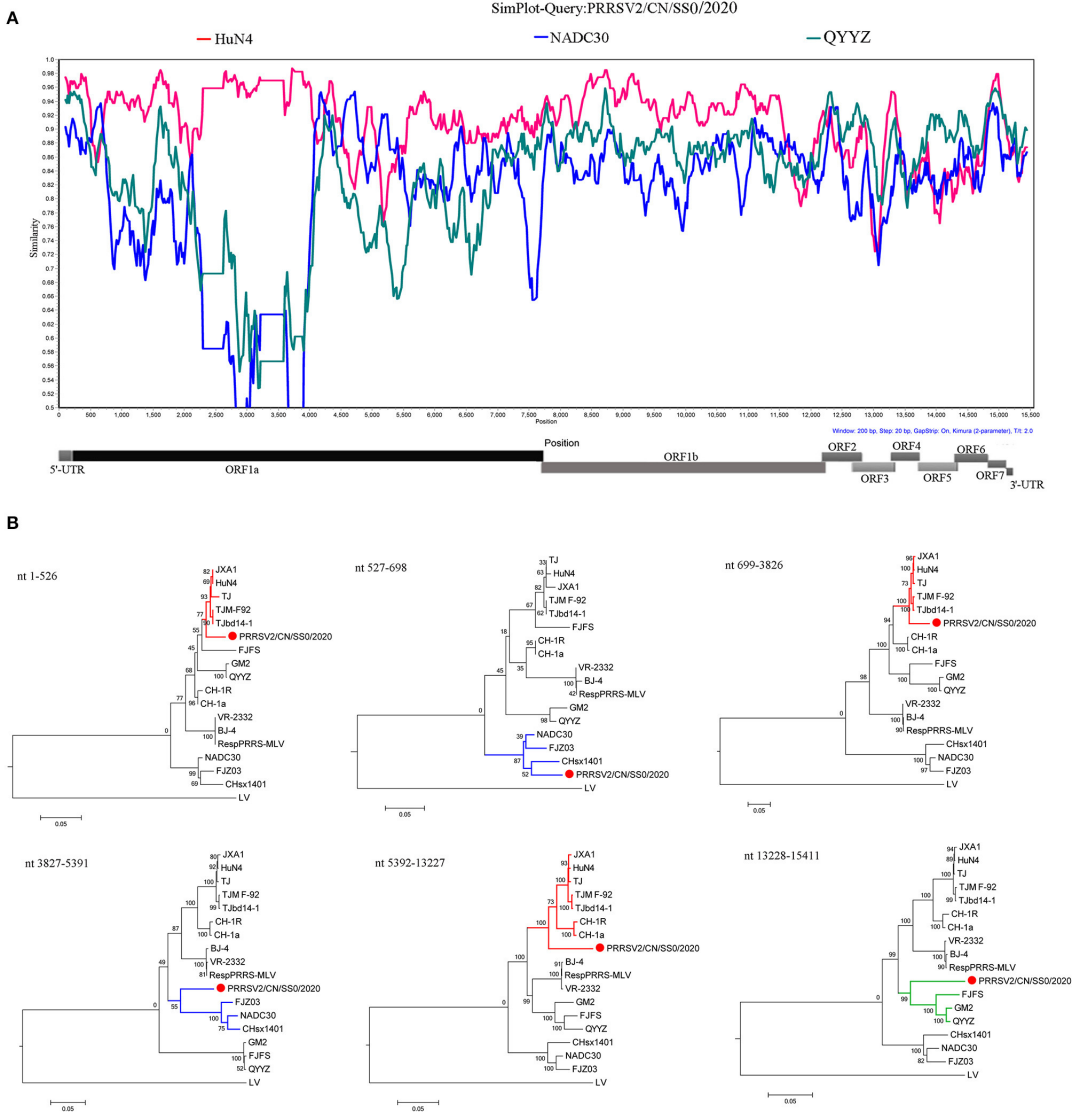
Similarity plot analysis **(A)** and phylogenetic trees analysis based on different regions **(B)** of PRRSV2/CN/SS0/2020. For similarity plot analysis, the y-axis indicates the percentage identity between the parental sequences and the query sequence. The complete genome RRSV2/CN/SS0/2020 was chosen as the query sequence, respectively.

The recombination analysis using the RDP version 4.10 software revealed that the four isolates are the result of recombination between JXA1-like, QYYZ-like, and NADC30-like strains circulating in China (Table 3, Figure 4, Supplementary Figure 2). Additionally, the putative recombinant events and statistically incongruent phylogenetic trees were further confirmed using SimPlot v3.5.1 and statistically incongruent phylogenetic trees (Figure 4, Supplementary Figure 2). From the similarity plot, the breakpoints of recombination events of four strains are mainly located in nsp1, nsp2, nsp3, nsp11, and nsp12 (Figure 4, Supplementary Figure 2).

**Table 3:** Information of recombination events of four isolates.

	**Breakpoint position**		**Major**	**Minor**
**Isolate**	**in alignment**		**parent**	**parent**	***P*** **-value**						
	**Beginning**	**Ending**			**RDP**	**GENECONV**	**Bootscan**	**MaxChi**	**Chimera**	**SiScan**	**3Seq**
PRRSV/CN/SS0/2020	526	698	HuN4	NADC30	2.707 × 10^−7^	–	4.288 × 10^−8^	2.716 × 10^−3^	5.675 × 10^−4^	–	8.854 × 10^−5^
	3,826	5,391	HuN4	NADC30	1.752 × 10^−4^	–	1.565 × 10^−15^	2.205 × 10^−6^	1.317 × 10^−8^	2.367 × 10^−17^	7.579 × 10^−12^
	11,788	15,411	HuN4	QYYZ	9.132 × 10^−24^	–	1.827 × 10^−21^	1.520 × 10^−28^	1.373 × 10^−9^	6.812 × 10^−8^	7.327 × 10^−14^
PRRSV/CN/SS1/2021	476	722	HuN4	NADC30	1.111 × 10^−10^	–	5.944 × 10^−11^	5.360 × 10^−5^	4.379 × 10^−7^	-	1.188 × 10^−9^
	1,837	2,211	HuN4	NADC30	1.071 × 10^−19^	–	4.382 × 10^−19^	3.864 × 10^−15^	3.221 × 10^−12^	1.002 × 10^−10^	3.730 × 10^−14^
	3,790	5,231	HuN4	NADC30	1.335 × 10^−42^	–	7.166 × 10^−39^	3.607 × 10^−24^	6.873 × 10^−24^	1.923 × 10^−24^	3.730 × 10^−14^
	13,227	15,411	HuN4	FJFS	3.905 × 10^−25^	–	1.803 × 10^−23^	5.300 × 10^−19^	8.432 × 10^−22^	1.320 × 10^−12^	1.865 × 10^−14^
PRRSV/CN/L3/2021	480	686	JXA1	NADC30	6.014 × 10^−12^	–	3.383 × 10^−12^	2.429 × 10^−06^	1.528 × 10^−07^	–	7.946 × 10^−11^
	1,845	5,390	JXA1	FJFS	2.715 × 10^−99^	1.571 × 10^−51^	2.248 × 10^−75^	4.195 × 10^−41^	1.632 × 10^−24^	2.000 × 10^−54^	1.332 × 10^−14^
	11,600	15,411	JXA1	NADC30	6.795 × 10^−21^	–	1.840 × 10^−19^	1.185 × 10^−18^	7.242 × 10^−22^	3.896 × 10^−13^	9.858 × 10^−13^
PRRSV2/CN/L4/2020	1,897	5,388	JXA1	NADC30	1.284 × 10^−94^	5.386 × 10^−53^	2.066 × 10^−88^	1.009 × 10^−41^	2.533 × 10^−47^	3.794 × 10^−48^	3.454 × 10^−14^
	11,401	15,411	JXA1	QYYZ	6.892 × 10^−21^	3.818 × 10^−04^	1.029 × 10^−14^	2.023 × 10^−06^	1.956 × 10^−09^	1.320 × 10^−13^	4.884 × 10^−14^

*Breakpoint position inPRRSV genome with reference to the VR-2332 strain*.

### PRRSV2/CN/SS0/2020 and PRRSV2/CN/L3/2021 Exhibited Pathogenicity for Pigs

**Figure 5:**
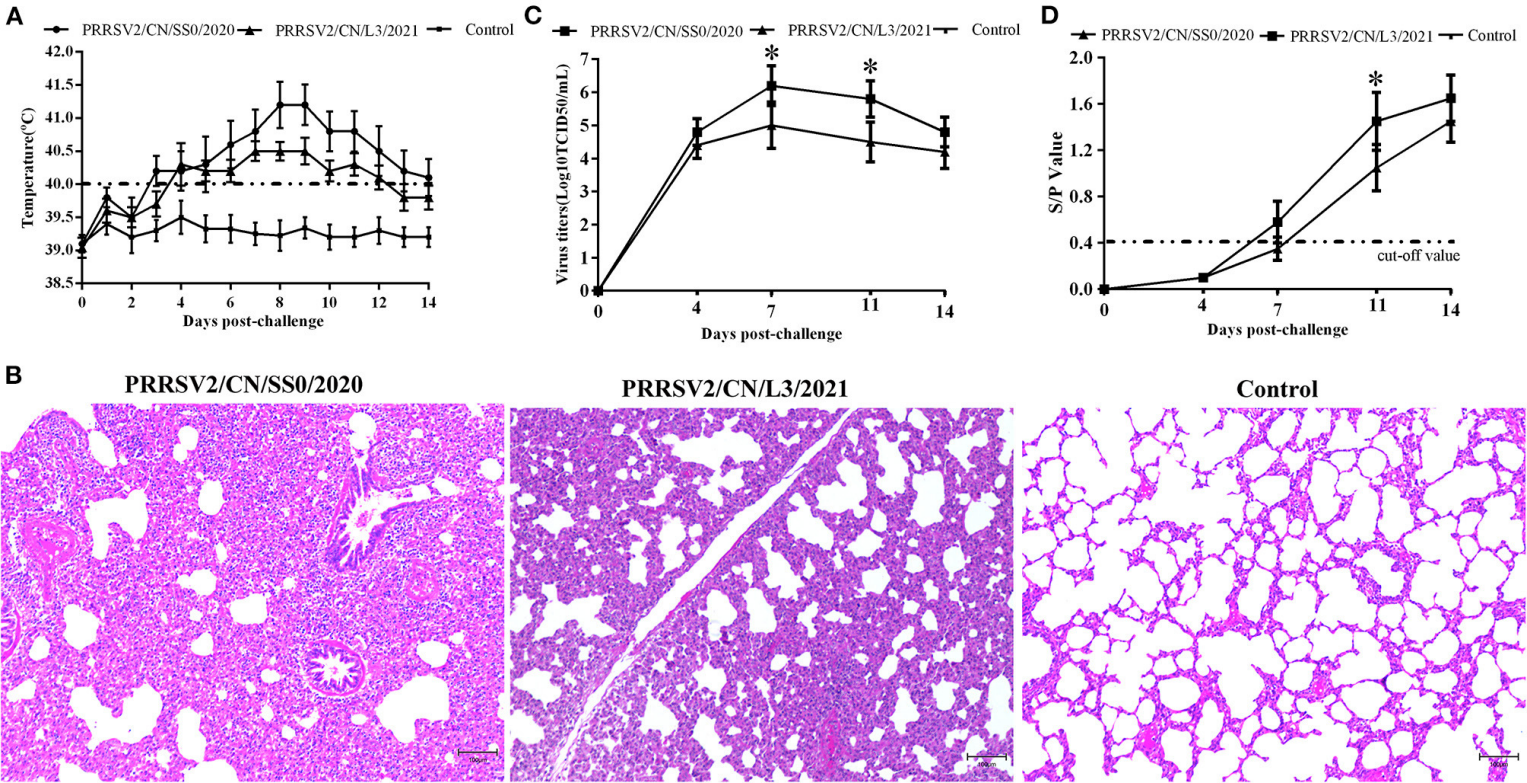
The rectal temperature, microscopic lung lesions, viremia, and antibody levels of pigs during the challenge experiment. **(A)** Rectal temperatures of pigs inoculated with PRRSV2/CN/SS0/2020, PRRSV2/CN/L3/2021, and DMEM. The clinical fever cut-off value was set at 40 °C. **(B)** Microscopic lung lesions of the inoculated pigs. **(C)** Dynamics of viral loads of the inoculated pigs during the experiment. **(D)** PRRSV-specific antibodies response in serum of challenged pigs. The cut-off value for seroconversion was set at a sample-to-positive (s/p) ratio of 0.4. Data are expressed as mean ± standard deviation (S.D). Asterisk indicates significant differences between the PRRSV2/CN/SS0/2020- and PRRSV2/CN/L3/2021-infected groups (*p < 0.05).

Pigs infected with PRRSV2/CN/SS0/2020 exhibited obvious clinical signs, including respiratory distress, anorexia, and coughing from 3 dpc. The body temperatures of PRRSV2/CN/SS0/2020-inoculated group began to reach above 40°C at 3–14 dpc, with a peak (>41 °C) at 8–9 dpc. In contrast, the pigs infected with PRRSV2/CN/L3/2021 had less fever and less severe clinical signs (Figure 5A). Control pigs showed normal rectal temperature and behavior throughout the experiment. One of five PRRSV2/CN/SS0/2020-infected pigs died at 10 dpc, whereas no mortality was observed in PRRSV2/CN/L3/2021-infected group and negative control group. In addition, pigs in PRRSV2/CN/SS0/2020-infected group had significantly more severe interstitial pneumonia than did pigs inoculated with PRRSV2/CN/L3/2021 (Figure 5B), which indicates that PRRSV2/CN/SS0/2020 is a highly virulent PRRSV isolate for pigs.

The PRRSV was isolated from the serum of pigs challenged with the two strains at 4 dpc. The mean virus titers of PRRSV2/CN/SS0/2020 or PRRSV2/CN/L3/2021 reached peak levels at 7 dpc (10^6.2^ TCID50/mL and 10^5.0^ TCID50/mL, respectively) and high viremia persisted until 14 dpc in every PRRSV-infected group (Figure 5C). Furthermore, the mean virus loads for PRRSV2/CN/SS0/2020-infected group were significantly higher than those of the PRRSV2/CN/L3/2021-infected group at 7 and 11 dpc (*p* < 0.05). Meanwhile, no PRRSV was detected in the control group throughout the experiment. The specific antibodies of PRRSV N protein in the sera of pigs were detected by a commercial ELISA kit. All pigs in the PRRSV2/CN/SS0/2020-infected group became seroconverted at 7 dpc, whereas PRRSV2/CN/L3/2021-infected were seroconverted at 11 dpc (Figure 5D). In contrast, PRRSV-specific antibodies were not detected in the control group throughout the experiment.

## Discussion

Porcine reproductive and respiratory syndrome virus is one of the most important pathogens causing substantial economic losses to the Chinese swine industry. According to the global PRRSV classification system, four different lineages of PRRSV (lineages 1, 3, 5, and 8) co-existed in Chinese swine herds, and two of them (lineages 1 and 8) are currently the predominant strains circulating in China. With the emergence of NADC30-like PRRSVs in China since 2012, recombinant strains have been frequently observed in the field. Notably, multiple studies have shown that PRRSV variants evolved from recombination events between lineage 1 and one or two other lineages (12–23). In this study, four PRRSV strains (PRRSV2/CN/SS0/2020, PRRSV2/CN/SS1/2021, PRRSV2/CN/L3/2021, and PRRSV2/CN/L4/2020) were also identified as recombinant viruses from three lineages of type 2 PRRSV (NADC30-like, QYYZ-like, and JXA1-like viruses). Importantly, the four strains in this study had a low whole-genome nucleotide similarity with other PRRSV sequences in GenBank (<92%), which raised the concern of the introduction of new viruses into other large-scale pig farms. Therefore, to further our knowledge about these recombinant PRRSV strains, we characterized them genetically and phylogenetically to obtain insights that link genotypic with phenotypic data.

The natural genomic recombination between different lineages of PRRSV has been shown to play an important role in the generation of novel strains (34–36). Several studies have shown that the patterns of recombination of PRRSV in the field are becoming increasingly complex; for instance, 14LY01-FJ, 14LY02-FJ, 15LY01-FJ, 15LY02-FJ, JL580, GXNN1839, GXYL1403, HeN1401, HeN1601, FJ1402, FJXS15, SC-d, and TJnh1501 have evolved from the recombination between two lineages (17, 18, 20, 22, 36–39), whereas SD17–38, PRRSV2/CN/N9185/2018, PRRSV2/CN/X4833/2018, SDhz1512, and SCcd16 were natural recombinant viruses among three lineages (12, 14, 16, 23). More surprisingly, FJLIUY-2017 was derived from recombinant strains of four lineages (15). Notably, the diversity of recombinant PRRSV strains poses a major obstacle to the effective control of viral transmission (38, 40). In the present study, a comparison of whole-genome sequences and recombination analysis revealed that the four recombinant isolates (PRRSV2/CN/L3/2021, PRRSV2/CN/L4/2020, PRRSV2/CN/SS02020, and PRRSV2/CN/SS1/2021) were also natural recombinant viruses among the three lineages (lineage 1, 3, and 8.7). However, full-length sequencing of the virus showed that the entire genome of the four strains had low identity with the PRRSV strains available in GenBank, suggesting that PRRSVs have undergone evolution *via* natural recombination in recent years. Previous studies showed that Nsp1, Nsp2, Nsp3, Nsp9, Nsp11, and ORF2 are the hot spots for PRRSV RNA recombination (12, 13, 15, 17, 22, 23, 25, 36). In the present study, breakpoints of the four recombinant strains were also mainly located in Nsp1, Nsp2, Nsp3, and Nsp11, indicating that PRRSV gains genetic diversity by increasing recombination events at specific regions.

Nsp2 and ORF5 are the most variable proteins in the viral genome and are usually used as target genes for the molecular epidemiological surveillance of PRRSV (27). Compared with VR2332, the Nsp2 gene of PRRSV contains different patterns of amino acid insertions and deletions. For example, an outbreak in China with highly pathogenic PRRSVs (HP-PRRSV, JXA1-like) in 2006 had a unique 30-aa deletion in the Nsp2 region, whereas a unique 131-aa (111aa+1aa+19aa) deletion pattern in Nsp2 is the footprint of NADC30 and NADC30-like strains (11, 41). In the present study, the Nsp2 gene of the two strains (PRRSV2/CN/L3/2021 and PRRSV2/CN/L4/2020) had the 131-aa deletion, which is identical to NADC30-like strains, suggesting that these two strains may belong to lineage 1. Additionally, 2-aa insertions were also found in PRRSV2/CN/L3/2021 and PRRSV2/CN/L4/2020. Previous studies showed that MLV TJM-F92 has a unique 150-aa deletion (aa481+aa537-566+aa628–747) signature in its Nsp2-coding region (30). Interestingly, two strains identified in our study (PRRSV2/CN/SS0/2020 and PRRSV2/CN/SS1/2021) had characteristic 150-aa deletions that were identical to TJbd14-1 (an MLV-like strain that evolved from TJM-F92) and TJM-F92, suggesting that the PRRSV genome may have evolved more compactly by eliminating dispensable genomic regions (42). Furthermore, the phylogenetic analysis based on the ORF5 gene demonstrated that the four strains belonged to the QYYZ-like virus. In addition, the GP5 antigenic regions in the four strains were similar to those of the related lineage 3 representative strains but different from other lineage representative strains. The discordance between Nsp2 patterns and ORF5 lineages resulted from the recombination of PRRSVs. In other words, there is limited understanding of the recombinant PRRSV genomic data, especially if quantifying PRRSV diversity has only focused on ORF5 and Nsp2 analysis. In addition, the 137th aa of the GP5 protein is assumed to differentiate the attenuated vaccine strain (A^137^) and the wild strain (S^137^). Residue S^137^ was present in all four strains, suggesting that the four strains may be wild viruses (43). Additionally, a retrospective survey found that a massive vaccination with HP-PRRSV live attenuated vaccine (TJM-F92) was effective in preventing and controlling PRRS in this farm since 2018. Thus, we propose that PRRSV2/CN/SS0/2020 and PRRSV2/CN/SS1/2021 may have evolved from recombination among MLV TJM-F92 and NADC30-like or other PRRSV strains. Whether this strain directly evolved from MLV TJM-F92 needs to be further investigated.

Viremia and severity of fever are closely related to PRRSV virulence (44). In the present study, PRRSV2/CN/SS0/2020-infected pigs had higher viremia than PRRSV2/CN/L3/2021 from 4 dpc to 14 dpc, suggesting that PRRSV2/CN/SS0/2020 has higher virulence for pigs. In addition, pigs inoculated with the PRRSV2/CN/SS0/2020 strain had persistently higher fever (>40°C for 12 days) and interstitial pneumonia compared to the PRRSV2/CN/L3/2021-infected group. Notably, one pig in the PRRSV2/CN/SS0/2020 group died within 2 weeks, whereas the pigs in the PRRSV2/CN/L3/2021 survived throughout the experiment. These data showed that PRRSV2/CN/SS0/2020 demonstrates higher pathogenicity than PRRSV2/CN/L3/2021. This finding is consistent with previous results that recombinant PRRSV strains derived from field PRRSV strains and vaccine-like strains could result in higher pathogenicity for pigs, such as FJXS15 and TJnh150 (13, 18).

In conclusion, we determined the complete genome sequences of four recombinant PRRSV isolates (PRRSV2/CN/SS0/2020, PRRSV2/CN/SS1/2021, PRRSV2/CN/L3/2021, and PRRSV2/ CN/L4/2020). Four isolates were natural recombinant among three lineages (lineage 1, 3, and 8) and have the absence of close relatives to PRRSV sequence in GenBank (<92%). PRRSV2/CN/SS0/2020 exhibits higher pathogenicity than PRRSV2/CN/L3/2021. Our findings suggest that PRRSVs have undergone genome evolution by recombination among field strains/MLV-like strains of different lineages.

## Data Availability Statement

The datasets presented in this study can be found in online repositories. The names of the repository/repositories and accession number(s) can be found in the article/Supplementary Material.

## Author Contributions

JLiu and CW performed the experiments. YX, CL, and YY conceived and designed the experiments. LL, CH, and JLi performed the data analyses. JLiu completed the writing of the manuscript. All authors contributed to the article and approved the submitted version.

## Funding

This study was supported by Leading Project Foundation of Science Department of Fujian Province (2021N0032), Research Foundation of Engineering Research Center for the Prevention and Control of Animal Original Zoonosis, Fujian Province University, (Grant No. 2021ZN002), Qimai Foundation of Shanghang District of Longyan City, Fujian Province (2020SHQM07) and Longyan University Young Talents Program.

## Conflict of Interest

The authors declare that the research was conducted in the absence of any commercial or financial relationships that could be construed as a potential conflict of interest.

## Publisher's Note

All claims expressed in this article are solely those of the authors and do not necessarily represent those of their affiliated organizations, or those of the publisher, the editors and the reviewers. Any product that may be evaluated in this article, or claim that may be made by its manufacturer, is not guaranteed or endorsed by the publisher.
