# Supplementary material for: Evolutionary Analysis of Four Recombinant Viruses of the Porcine Reproductive and Respiratory Syndrome Virus From a Pig Farm in China
Source: Front Vet Sci. 2022 Jun 24;9:933896. doi: 10.3389/fvets.2022.933896 (PMC9270021; doi:10.3389/fvets.2022.933896)
Supplement: Supplementary file 1 [file Table_1.DOCX]

**Supplementary Figure 1**

**
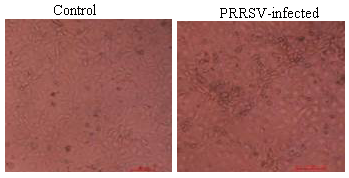
**

Supplementary FIGURE 1 **CPE of PRRSV infected MARC-145 cells at 72 h post-inoculation.**

**Supplementary FIGURE 2**


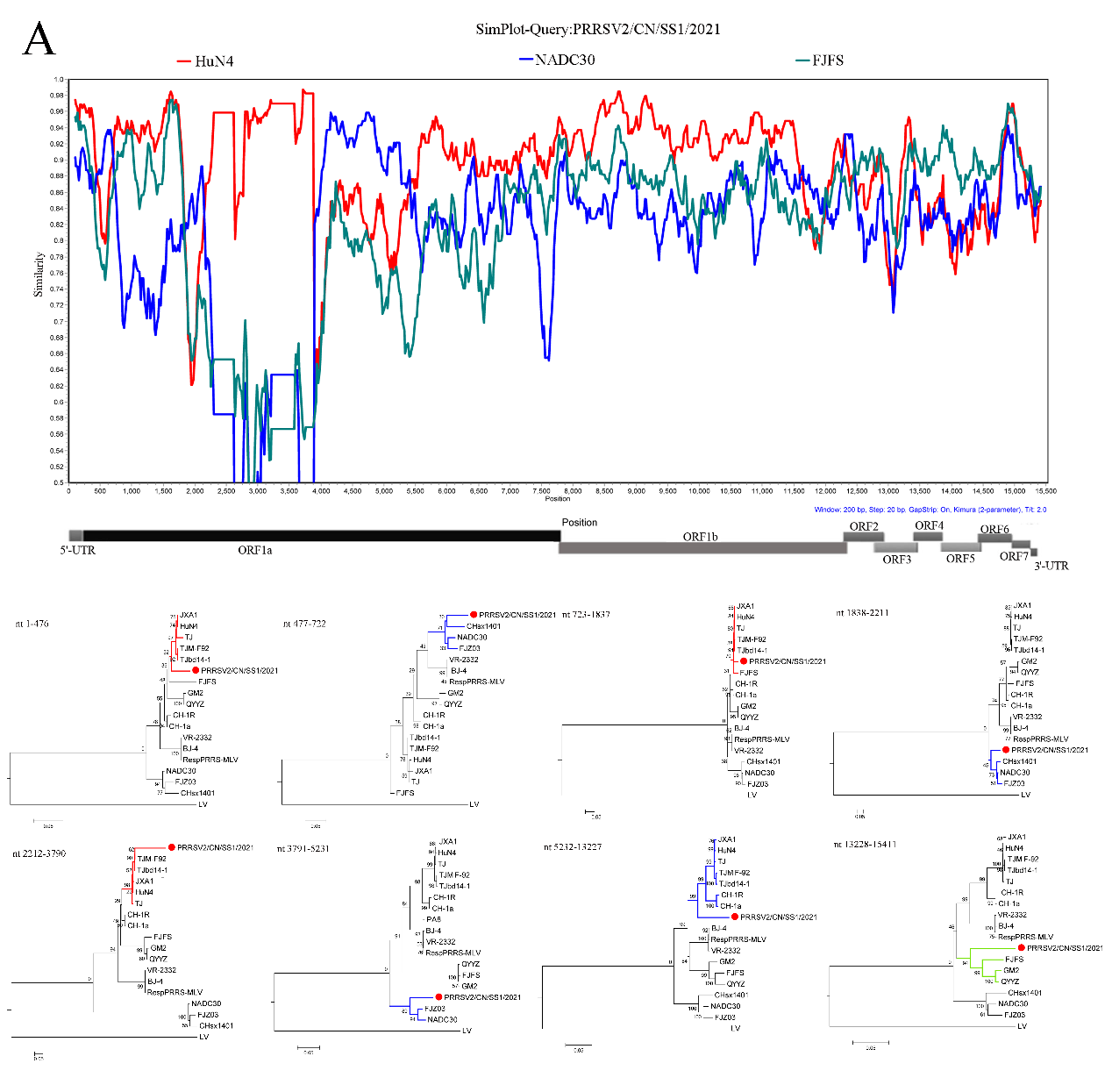

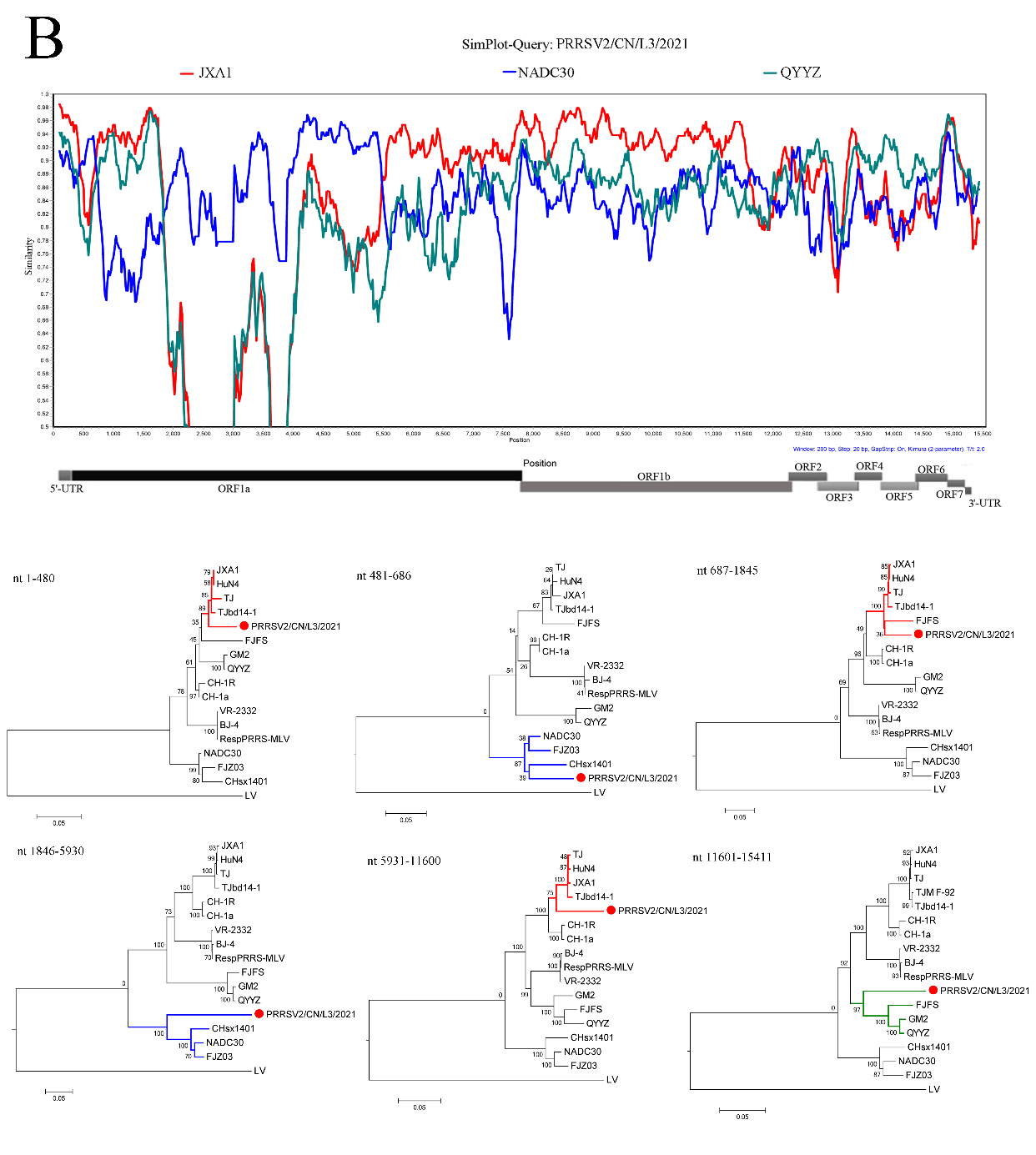

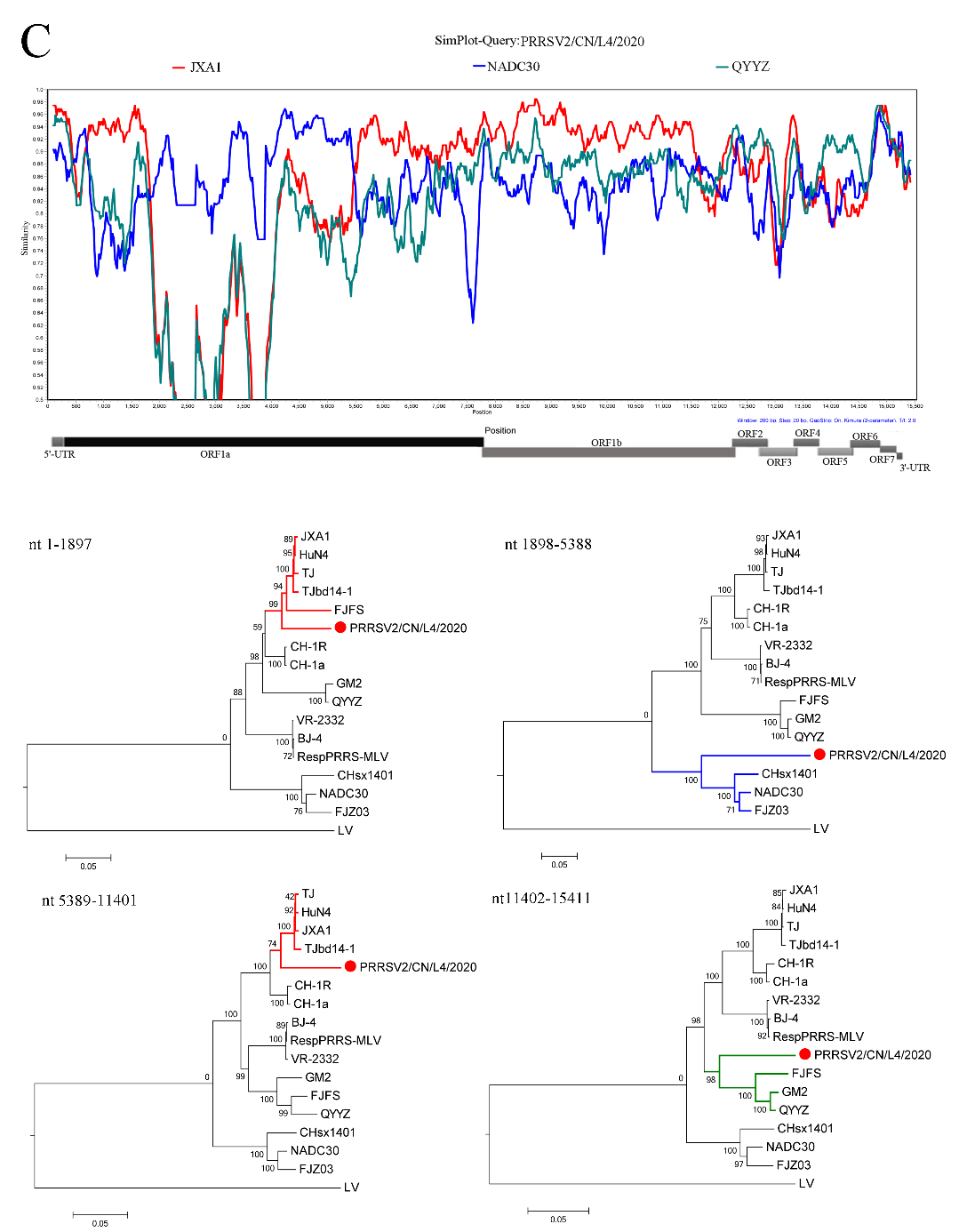


Supplementary FIGURE 2 **Similarity plot analyze and phylogenetic trees analyze based on different regions of PRRSV2/CN/SS1/2021 (A), PRRSV2/CN/L3/2021 (B) and PRRSV2/CN/L4/2020 (C)**. For Similarity plot analysis, the y-axis indicates the percentage identity between the parental sequences and the query sequence. The complete genome PRRSV2/CN/SS1/2021, PRRSV2/CN/L3/2021 and PRRSV2/CN/L4/2020 was chosen as query sequence, respectively.
