# Supplementary material for: Evolutionary Analysis of Four Recombinant Viruses of the Porcine Reproductive and Respiratory Syndrome Virus From a Pig Farm in China
Source: Front Vet Sci. 2022 Jun 24;9:933896. doi: 10.3389/fvets.2022.933896 (PMC9270021; doi:10.3389/fvets.2022.933896)

**Supplementary Table 1 List of primers used in this study**

| Fragment | Sequence(5'-3') | Location^a^ | Length of PCR products (bp) |
| --- | --- | --- | --- |
| A | GAATTCATGACGTATAGGTGTTGGCT | 1-3022 | 3022 |
|  | AGCATGTCCACCCTRTCCCAC |  |  |
| B | CCTCYTTTGATTGGRAYGTTGTG | 2191-6192 | 4002 |
|  | TCCGRGGCARGAADGCATA |  |  |
| C | CAAGTYCTRATGATCAGRCTTCT | 6024-9083 | 3060 |
|  | TRTCCATDAGTATTGCAGCHG |  |  |
| D | GCAGRTGCCTYGARGCYGAT | 8463-11604 | 3142 |
|  | TAAATTTTCCCTTTCTGDCGYGC |  |  |
| E | AGGACTGGGAGGAYTAYAAT | 11549-14390 | 2841 |
|  | CGGACGACAAATGCGTGGTTAT |  |  |
| F | GATAACCACGCRTTYGTCGT | 14367-15020 | 654 |
|  | TATAGCGGCCGCTAATTWCGGCC |  |  |

a position in genome of PCR products with respect to the NADC30 genome.

**Supplementary Table 2 Comparison of the full-length genomes of 4 PRRSV strains in this study**


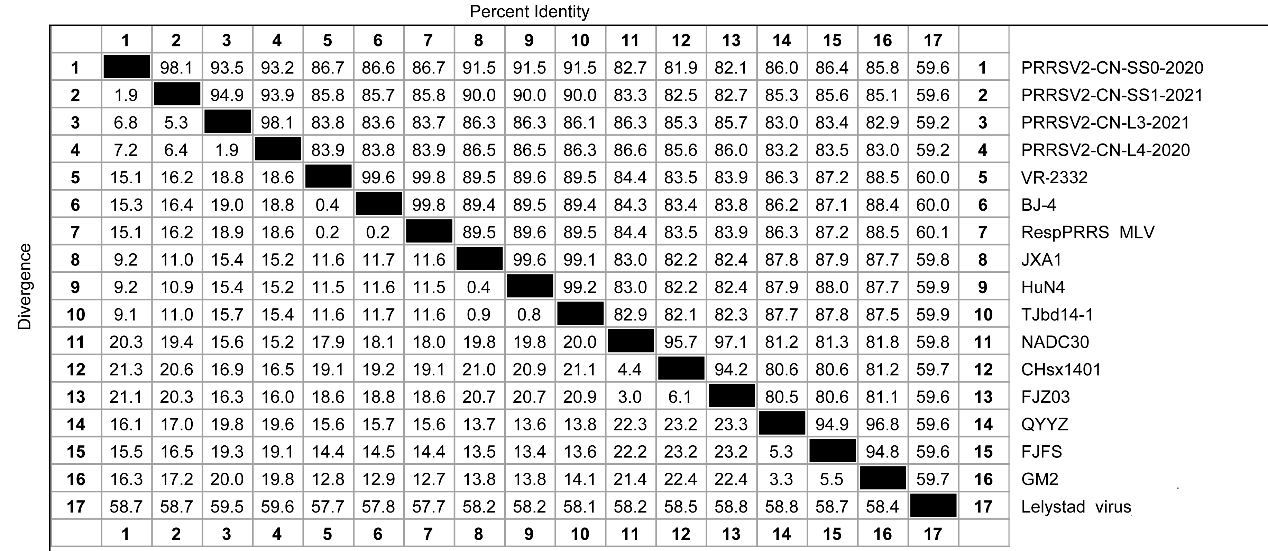

Supplement: Supplementary file 2 [file Table_2.DOCX]
